# Developmental origin of postnatal cardiomyogenic progenitor cells

**DOI:** 10.4155/fsoa-2016-0006

**Published:** 2016-03-29

**Authors:** Yuan-Hung Liu, Ling-Ping Lai, Shih-Yun Huang, Yi-Shuan Lin, Shinn-Chih Wu, Chih-Jen Chou, Jiunn-Lee Lin

**Affiliations:** 1 Section of Cardiology, Cardiovascular Center, Far Eastern Memorial Hospital, Pan Chiao, New Taipei City, Taiwan; 2 Graduate Institute of Clinical Medicine, College of Medicine, National Taiwan University, Taiwan; 3 College of Informatics, Yuan Ze University, Chungli, Taoyuan, Taiwan; 4 Department of Internal Medicine, National Taiwan University Hospital, Taipei, Taiwan; 5 Department of Animal Science & Technology, National Taiwan University, Taipei, Taiwan

**Keywords:** cardiac regeneration, cardiac progenitor cells, *Nkx2.5*, myocardial infarction, epicardium-derived cells

## Abstract

**Aim:**

To trace the cell origin of the cells involved in postnatal cardiomyogenesis.

**Materials & methods:**

Nkx2.5 enhancer-eGFP (Nkx2.5 enh-eGFP) mice were used to test the cardiomyogenic potential of Nkx2.5 enhancer-expressing cells. By analyzing Cre excision of activated Nkx2.5-eGFP+ cells from different lineage-Cre/Nkx2.5 enh-eGFP/ROSA26 reporter mice, we traced the developmental origin of Nkx2.5 enhancer-expressing cells.

**Results:**

Nkx2.5 enhancer-expressing cells could differentiate into striated cardiomyocytes both *in vitro* and *in vivo*. Nkx2.5-eGFP+ cells increased remarkably after experimental myocardial infarction (MI). The post-MI Nkx2.5-eGFP+ cells originated from the embryonic epicardial cells, not from the pre-existing cardiomyocytes, endothelial cells, cardiac neural crest cells or perinatal/postnatal epicardial cells.

**Conclusion:**

Postnatal Nkx2.5 enhancer-expressing cells are cardiomyogenic progenitor cells and originate from embryonic epicardium-derived cells.

## Abstract (lay abstract)

**Lay abstract:** Recent studies report that postnatal mammalian hearts undergo cardiomyocyte refreshment; however, evidence is lacking for the cell origin of the cells involved in postnatal cardiomyogenesis. In this study, we confirmed that Nkx2.5 cardiac progenitor cells existed in the postnatal mouse heart and could differentiate into striated cardiomyocytes both *in vitro* and *in vivo*. The developmental origin of these postnatal Nkx2.5 cardiac progenitor cells are from the embryonic epicardial cells.

**Figure 1.**
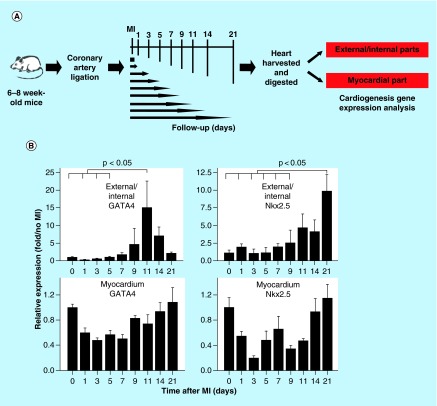
**Myocardial injury triggers cardiogenesis gene expression.** **(A)** Schematic diagram of the analysis of cardiogenesis gene expression after myocardial injury. The hearts were harvested from the mice before surgery and 1, 3, 5, 7, 9, 11, 14 and 21 days following MI. **(B)** Gene expression of the hearts before and after MI. In the external and internal parts of the hearts, expression of the cardiogenesis genes *GATA4* and *Nkx2.5* increased after MI. *GATA4* expression peaked on day 11, and *Nkx2.5* expression peaked on day 21 post MI (n = 4 to 5 in each group). MI: Myocardial injury.

**Figure 2.**
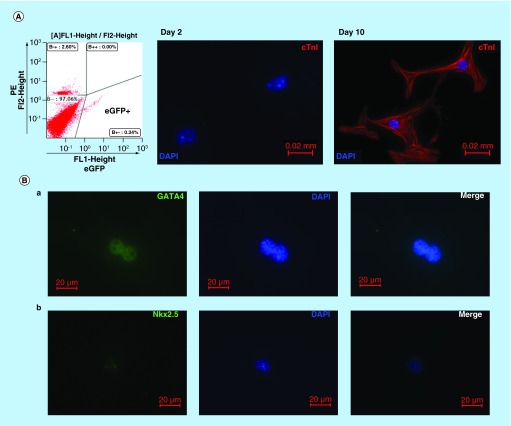
**Cardiomyogenic potential of Nkx2.5 enhancer-expressing cells.** **(A)** Nkx2.5 enh-eGFP^positive^ cells sorted from the heart of postnatal Nkx2.5 enh-eGFP mice were cultured to test the cardiomyogenic potential of postnatal Nkx2.5 enhancer-expressing cells. Initially post sorting (2 days later), Nkx2.5 enh-eGFP^positive^ cells stained negative for cTnI. After culture for 10 days, isolated Nkx2.5 enh-eGFP^positive^ cells differentiated into cTnI+ cardiomyocytes with striation. **(B)** Immunostaining of activated Nkx2.5 enh-eGFP^positive^ cells with precardiac mesoderm marker (GATA4) and cardiac precursor marker (Nkx2.5) from the hearts of Nkx2.5 enh-eGFP mice 1 week following MI. The eGFP+ cells stained positive for GATA4 and Nkx2.5.

**Figure 3.**
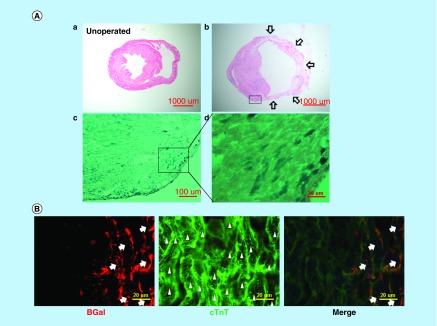
***In vivo* evidence that Nkx2.5 enhancer-expressing cells differentiate into cardiomyocytes.** **(A)** Lineage tracing of Nkx2.5 enhancer-expressing cells after MI. Representative LacZ-stained heart sections from the inducible Nkx2.5 enh-Cre/R26R-LacZ mice with tet-off system. **(a)** Unoperated group, treated with doxycycline from conception to sacrifice. **(b)** 6 weeks post MI and treated with doxycycline from conception to MI. Note some cells in the subepicardium and myocardium stained positive for LacZ. Arrows indicate infarct area. **(c)-(d)** Higher magnification of **(b)**. **(B)** Immunostaining of representative heart sections from the inducible Nkx2.5 enh-Cre/R26R-LacZ mice, 6 weeks post MI and treated with doxycycline from conception to MI. The descendent cells of activated Nkx2.5 enhancer-expressing cells (red fluorescence; arrows, labeled by anti-β-galactosidase antibody, BGal) expressed cardiomyocyte marker cardiac troponin T (cTnT) (green fluorescence; arrow heads). Cell nuclei visualized with DAPI staining.

**Figure 4.**
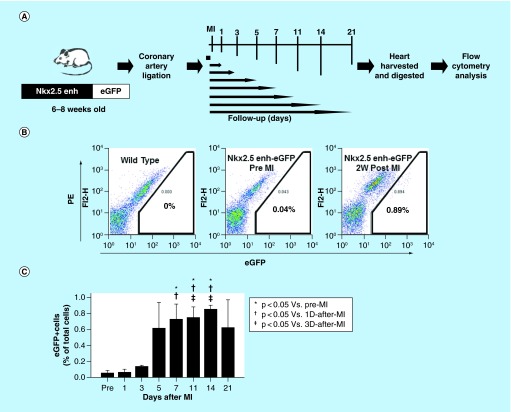
**Expansion of Nkx2.5 enh-eGFP^positive^ cells after experimental MI.** **(A)** Schematic diagram of the analysis of Nkx2.5 enh-eGFP^positive^ cells after coronary ligation. **(B)** Flow cytometry of the percentage of Nkx2.5 enh-eGFP^positive^ cells before and 2 weeks following MI. The baseline cell percentage of Nkx2.5 enh-eGFP^positive^ cells was low, but increased markedly by 2 weeks following MI. **(C)** Quantification of the percentage of Nkx2.5 enh-eGFP^positive^ cells in the mice before and 1, 3, 5, 7, 11, 14 and 21 days following MI. The Nkx2.5 enh-eGFP^positive^ cells increased after surgery and were activated up to 14-fold 2 weeks following MI (n = 4 to 5 in each group).

**Figure 5.**
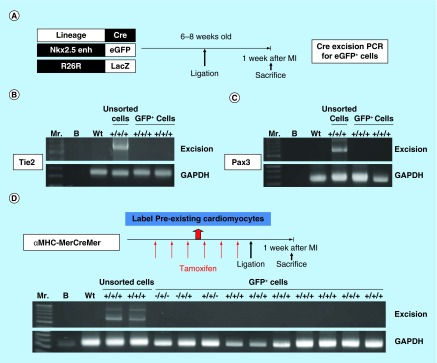
**Lineage tracing of the postnatal Nkx2.5 cardiac progenitor cells.** **(A)** The strategy used to analyze the developmental origin of activated postnatal Nkx2.5 cardiac progenitor cells by excision PCR on sorted Nkx2.5 enh-eGFP+ cells from the hearts of different lineage-Cre/Nkx2.5 enh-eGFP/R26R-LacZ mice. **(B)** Excision PCR of the activated Nkx2.5 enh-eGFP^positive^ cells from the hearts of endothelial cell lineage, Tie2-Cre/Nkx2.5-eGFP/R26R-LacZ mice. The positive control was the DNA from the unsorted cells of triple transgenic mouse heart. The negative excision bands of the sorted eGFP+ cells confirmed that the activated Nkx2.5 enh-eGFP^positive^ cells following MI did not originate from the endothelial cells. **(C)** Excision PCR of the activated Nkx2.5 enh-eGFP^positive^ cells from the hearts of cardiac neural crest cell lineage, Pax3-Cre/Nkx2.5-eGFP/R26R-LacZ mice. The result showed that the activated Nkx2.5 enh-eGFP^positive^ cells did not derive from the cardiac neural crest cells. **(D)** Excision PCR of the activated Nkx2.5 enh-eGFP^positive^ cells from the hearts of cardiomyocyte lineage, αMHC-MerCreMer/Nkx2.5 enh-eGFP/R26R-LacZ mice. Pre-existing cardiomyocytes were labeled with 4-OH tamoxifen prior to MI. The results showed that the activated Nkx2.5 enh-eGFP^positive^ cells following MI did not originate from the pre-existing cardiomyocytes.

**Figure 6.**
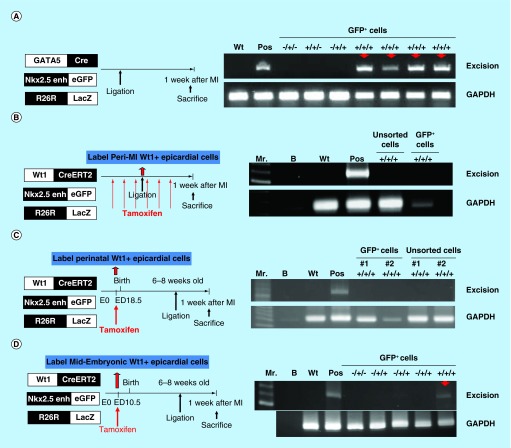
**Postnatal cardiac progenitor cells originate from the embryonic epicardial cells.** **(A)** Excision PCR of the activated Nkx2.5 enh-eGFP^positive^ cells from the hearts of epicardial cell lineage, GATA5-Cre/Nkx2.5 enh-eGFP/R26R-LacZ mice. The positive excision bands (arrows) of the sorted Nkx2.5 enh-eGFP^positive^ cells suggested the epicardial origin of postnatal Nkx2.5 cardiac progenitor cells. **(B)** Excision PCR of the activated Nkx2.5 enh-eGFP^positive^ cells from the hearts of adult epicardial lineage, Wt1^CreERT2^/Nkx2.5 enh-eGFP/R26R-LacZ mice. The peri-MI Wt1 cells were labeled with 4-OH tamoxifen before and after MI. The result showed that the activated Nkx2.5 enh-eGFP^positive^ cells did not arise from the peri-MI Wt1 epicardial cells. **(C)** Excision PCR of the activated Nkx2.5 enh-eGFP^positive^ cells from the hearts of perinatal epicardial lineage, Wt1^CreERT2^/Nkx2.5 enh-eGFP/R26R-LacZ mice, with labeled perinatal Wt1 cells via 4-OH tamoxifen injection on ED 18.5. The results showed that the activated Nkx2.5 enh-eGFP^positive^ cells did not derive from the perinatal Wt1 epicardial cells. **(D)** Excision PCR of the activated Nkx2.5 enh-eGFP^positive^ cells from the hearts of embryonic epicardial lineage, Wt1^CreERT2^/Nkx2.5 enh-eGFP/R26R-LacZ mice, labeled the embryonic Wt1 cells with 4-OH tamoxifen injection on ED 10.5. The positive excision band (arrow) of the sorted Nkx2.5 enh-eGFP^positive^ cells suggestive of the embryonic epicardial origin of postnatal Nkx2.5 cardiac progenitor cells.

**Figure 7.**
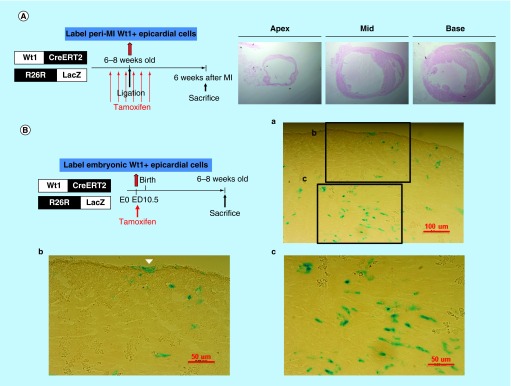
**LacZ staining of heart sections from the Wt1^CreERT2^/R26R-LacZ mice.** **(A)** Peri-MI Wt1 cells were labeled with 4-OH tamoxifen for 1 week prior to MI and for 1 week after MI. No LacZ+ cells were noted, indicating that very few Wt1 epicardial cells existed in the adult heart, even after myocardial injury. **(B)** LacZ staining of the representative heart sections from the 6- to 8-week-old Wt1^CreERT2^/R26R-LacZ mice labeled the embryonic Wt1 cells with 4-OH tamoxifen injection on ED 10.5. Note the epicardial cells (arrow head) and other cardiac cells were labeled LacZ positive.

The mammalian heart is thought to be a terminally-differentiated organ with lack of regeneration due to its very limited ability for self repair. By contrast, some simple vertebrates, such as zebrafish, display almost full cardiac regeneration after injury [1,2]. Emerging evidence suggests postnatal mammalian hearts undergo cardiomyocyte renewal [3–8].

By measuring ^14^C in human cardiomyocytes, Bergmann *et al*. estimated that the cardiomyocyte renewal rate in humans is about 1%/year at the age of 25 years, and that rate decreases to 0.45%/year by the age of 75 years [3]. A recent mouse study estimated an annual renewal rate of cardiomyocytes 0.76%/year [7]. However, the cell origin contributing to postnatal cardiomyocyte renewal remains debated. Using N-15 to label dividing cell DNA and 4-OH tamoxifen to label pre-existing cardiomyocytes of αMHC-MerCreMer/ZEG mice GFP, Senyo *et al*. demonstrated that following MI, 71% of N-15+ cardiomyocytes were labeled. Those authors interpreted the data as mammalian heart renewal by pre-existing cardiomyocytes because most of the newly forming cardiomyocytes (N-15+ cardiomyocytes) were from the pre-existing cardiomyocytes (GFP+ cardiomyocytes) [7]. However, 4-OH tamoxifen labeled 80% of the pre-existing cardiomyocytes, suggesting a higher percentage of the new-forming cardiomyocytes originated from stem/progenitor cells. Hsieh *et al*. previously reported that stem cells refresh mammalian cardiomyocytes in mice post-injury based on indirect pulse-chase evidence [4]. Some other evidence also shows that cardiomyocytes may arise from progenitor or stem cells [9–13].

Cardiac progenitor cells with different surface markers, such as c-Kit^+^ cells [9], Sca-1^+^ cells [10], and side population cells (ATP-binding cassette transporter expressing cells) [11], have been identified in postnatal mammalian hearts, and all of those cells are able to differentiate into cardiomyocytes *in vitro*. Cardiosphere-derived cells, which have been isolated from the adult mammalian hearts and are a heterogeneous collection of cells, also reportedly differentiate into cardiomyocytes [14,15]. Myocardial progenitors (Wt1 progenitors) in the epicardial layer of mouse hearts are reported to form cardiomyocytes within the damaged heart [12]. Chong *et al*. also found a population of adult cardiac-resident mesenchymal stem cells with multilineage differentiation potential [13]. However, the markers used to identify those populations of cells were neither tissue- nor cardiac lineage-specific and the origin of those progenitors remains unclear.

*Nkx2.5*, a homeodomain-type transcription factor, is one of the earliest transcription factors expressed during embryonic cardiogenesis. *Nkx2.5* is required for terminal differentiation and morphogenesis of the early developing heart [16,17]. Using Nkx2.5 enh-eGFP mice that express eGFP only in Nkx2.5 progenitor cells but not in cardiomyocytes, Wu *et al*. documented the ability of Nkx2.5-enh-eGFP cells in the developing hearts to undergo bipotential differentiation into cardiomyocytes and smooth muscle cells [18]. The finding that perinatal loss of *Nkx2.5* leads to conduction and contraction defects indicates the importance of *Nkx2.5* in the postnatal cardiac development [19].

The aim of this study was to determine if the postnatal Nkx2.5 enhancer expressing cells are cardiomyogenic progenitor cells and to trace the developmental origin of these progenitor cells.

## Materials & methods

### Animals

This study conformed with the Guide for the Care and Use of Laboratory Animals published by the United States National Institutes of Health (NIH Publication No. 85–23, revised 1996). The Nkx2.5 enh-eGFP mice [18], inducible Nkx2.5 enh-Cre mice [20] and GATA5-Cre [21] mice were generously supplied by Sean Wu, Stanford University School of Medicine. The α-myosin heavy chain-MerCreMer (αMHC-MerCreMer) [22], Tie2-Cre [23], Pax3-Cre [24], Wt1^CreERT2^ [25] and R26R-LacZ [26] mice were obtained from the Jackson Laboratory (CA, USA). C57BL/6J mice were obtained from National Laboratory Animal Center in Taiwan. Lineage-Cre/Nkx2.5 enh-eGFP mice were created by breeding αMHC-MerCreMer, Tie2-Cre, Pax3-Cre, GATA5-Cre or Wt1^CreERT2^ mice with Nkx2.5 enh-eGFP mice. R26R-LacZ mice were used as reporter mice. The purposes of the genetically manipulated mice are summarized in Table 1. All animal experiments were approved by Institutional Animal Care and Use Committee at the Far Eastern Memorial Hospital, New Taipei City, Taiwan (approval number: 99-1-47, 101-1-01, 102-02-07-A, 102-02-16-A).

### Surgery

MI was created by permanent ligation of the left anterior descending coronary artery approximately 2 mm beneath the left atrial appendage after the mice were anesthetized via intraperitoneal injection of a combination of ketamine (100 mg/kg) and xylazine (10 mg/kg), intubated with ventilator support, and underwent left thoracotomy.

### Gene expression determination by RT-qPCR

The hearts from the mice were dissected and digested with collagenase solution (collagenase A, 10 mg/ml and collagenase B, 10 mg/ml [both from Roche Diagnostics] in 10 mM HEPES (Sigma-Aldrich) buffered solution in 20% fetal calf serum) at 37°C. The external (epicardial/subepicardial) and internal (endocardial/subendocardial) parts of the heart were obtained by digesting the whole heart with collagenase for 1 h and the myocardial part was obtained from trituration and digestion of the remaining heart tissue. Cells from digested hearts were lysed with Trizol (Invitrogen, CA, USA). Total RNA was purified and stored at -80°C. cDNA was generated using a SuperScript III (Invitrogen) synthesis kit. Reverse transcription-quantitative polymerase chain reaction (RT-qPCR) was performed using Roche LightCycler 480II System (Roche Diagnostics, IN, USA) for 40 cycles. The primers were: *GATA4*, forward: 5′-TCT CAC TAT GGG CAC AGC AG-3′, reverse: 5′- CGA GCA GGA ATT TGA AGA GG-3′; *Nkx2.5*, forward: 5′- GCT ACA AGT GCA AGC GAC AG-3′, reverse: 5′- GGG TAG GCG TTG TAG CCA TA -3′.

### Differentiation of postnatal Nkx2.5 enh-eGFP+ cells into cardiomyocytes

After the postnatal Nkx2.5 enh-eGFP mice were euthanized with CO_2_, the hearts were minced and dissociated using collagenase solution at 37°C. The cells were suspended in differentiation medium containing IMDM, 20% fetal calf serum, 5000 IU/ml penicillin/streptomycin, 200 mM L-glutamine (all from Gibco-Invitrogen, NY, USA), 1.5 × 10^-4^M 1-thioglycerol (Sigma-Aldrich), and 50 μg/ml ascorbic acid (Sigma-Aldrich) [18]. The eGFP^positive^ live cells were isolated using FACS on a MoFlo XDP sorter (Beckman Coulter, CA, USA) and cultured onto fibronectin (Sigma-Aldrich, MO, USA)-coated chamber slides in differentiation medium and incubated in a humidified incubator at 37°C and 5% CO_2_ for 10 days to study the cardiomyogenic differentiation potential of the postnatal Nkx2.5 enh-eGFP+ cells.

### Flow cytometry & FACS sorting

After the mice were euthanized with CO_2_, the hearts from the C57BL/6J mice, Nkx2.5 enh-eGFP mice, as well as the lineage-Cre (αMHC-Cre, αMHC-MerCreMer, Tie2-Cre, Pax3-Cre, GATA5-Cre, Wt1^CreERT2^)/Nkx2.5-enh-eGFP/R26R-reporter (R26R-Tomato or R26R-LacZ) mice were dissected and digested for flow cytometry and FACS sorting. Following dissection, the hearts were immediately digested with collagenase solution. A single-cell suspension was obtained by triturition, followed by filtration with cell strainer. The eGFP^positive^ live cells, identified by negative propidium iodine staining, were isolated using FACS on a MoFlo XDP sorter (Beckman Coulter) and cultured in differentiation medium. Flow cytometry data were acquired by BD FACSCalibur (BD Biosciences, CA, USA) and processed by FlowJo v7.5 software (TreeStar, OR, USA).

### Labeling of the pre-existing cardiomyocytes

4-OH tamoxifen (Sigma-Aldrich) in DMF (1 mg/10 μl) was dissolved in sunflower oil at a concentration of 10 mg/ml then injected intraperitoneally into αMHC-MerCreMer/Nkx2.5 enh-eGFP/R26R-Tomato or R26R-LacZ mice at a dose of 2 mg three-times a week for 2 weeks (i.e., a total of 12 mg) to label the pre-existing cardiomyocytes with either red fluorescence or as β-galactosidase+.

### Labeling variant stages of epicardial Wt1 cells

4-OH tamoxifen in DMF (1 mg/10 μl) was dissolved in sunflower oil at a concentration of 10 mg/ml then injected intraperitoneally into Wt1^CreERT2^/Nkx2.5 enh-eGFP/R26R-LacZ mice. The labeling of peri-MI Wt1 cells was by administering 4-OH tamoxifen at a dose of 2 mg injected intraperitoneally three-times a week for 1 week prior to MI and three-times a week for 1 week after MI (i.e., a total of 12 mg) to label the adult epicardial Wt1 cells β-galactosidase+. The labeling of perinatal Wt1 cells was achieved by administering 2 mg of 4-OH tamoxifen intraperitoneally into the dams on embryonic day (ED) 18.5. The embryonic Wt1 cells were labeled with 1 mg 4-OH tamoxifen on ED 10.5 in combination with 1 mg/kg/day progesterone on ED 10.5–11.5.

### Immunohistochemistry & immunofluorescence

After the mice were euthanized with CO_2_, freshly isolated hearts were harvested, washed with phosphate buffered solution (PBS), incubated in 30% sucrose in PBS overnight, cryo-embedded, cut into 10-μM thick cross-sections and fixed with 4% paraformaldehyde (Sigma-Aldrich) in PBS. The heart sections of Nkx2.5 enh-eGFP transgenic mice were stained with the primary antibody against GFP (1:200, Invitrogen), a biotin-SP-conjugated secondary antibody (Jackson ImmunoResearch, PA, USA) and Streptavidin-Alexa Fluor^®^ 488 conjugate (Invitrogen) for visualization. The heart sections of inducible Nkx2.5 enh-Cre/R26R-LacZ mice were stained with antibodies specific for β-galactosidase (1:200, 55976, rabbit polyclonal IgG, MP Biomedicals, CA, USA) and cardiac troponin T (cTnT) (1:200, sc-8121, goat polyclonal IgG, Santa Cruz, TX, USA) using Alexa Fluor conjugated secondary antibody (Invitrogen) for visualization. For β-galactosidase staining (LacZ staining), freshly dissected mouse hearts were prepared as described above and incubated at 37°C in 1 mg/ml X-gal substrate (Thermo Fisher Scientific, MA, USA). The X-gal stained sections were then counterstained with Nuclear Fast Red. Cultured cells were fixed with 4% paraformaldehyde in PBS and stained with antibody specific for Nkx2.5 (1:200, Santa Cruz), GATA4 (1:200, Santa Cruz), and cardiac troponin I (cTnI) (1:200, sc-8118, goat polyclonal IgG, Santa Cruz) using Alexa Fluor 594-conjugated secondary antibody (A-21468, chicken antigoat polyclonal IgG, Invitrogen) for visualization.

### Excision PCR

The eGFP^positive^ cells from the digested hearts of variant lineage-Cre/Nkx2.5 enh-eGFP/R26R-LacZ: ±/+/± mice were sorted and cultured in differentiation medium. Cellular DNA was isolated using the Univers All Extraction kit (Yeastern Biotech, Taipei, Taiwan) and analyzed by PCR for the presence of excision. The primers were: forward: 5′-TGG CTT ATC CAA CCC CTA GA-3′, and reverse: 5′-GTT TTC CCA GTC ACG ACG TT-3′. GAPDH was used as the control.

### Statistical analysis

Numerical data are presented as mean ± standard deviation in the text and as mean ± standard error of the mean in the figures. Statistical analysis was performed using two-tailed t-test to compare the mean of two groups, and using ANOVA with Bonferroni's *post hoc* to make multiple comparisons. Statistical analysis was performed using SPSS 13.0 for Windows software (SPSS Inc., IL, USA). Probability values of p < 0.05 were considered statistically significant.

## Results

### Myocardial injury triggers cardiogenesis gene expression

To determine if myocardial injury triggers cardiogenesis, we did coronary artery ligation and sham operation on 6- to 8-week-old C57BL/6J mice. The hearts were harvested from the mice 0, 1, 3, 5, 7, 9, 11, 14 and 21 days after MI (n = 4 to 5 in each group) (Figure 1A). Because studies in zebrafish have shown that cardiac injury activates the epicardial cell layer and initiates cardiac regeneration at the subepicardial layer [27,28], differential gene expression analysis was performed. The external (epicardial/subepicardial) and internal (endocardial/subendocardial) parts of the heart were obtained by digesting the whole heart with collagenase for 1 h and the myocardial part was obtained from trituration and digestion of the remaining heart tissue. At present, there are no good and standard methods to separate epicardial layers from the myocardium. We therefore used collagenase digestion method to separate epicardium from myocardium. In theory, cells in the outer layers (e.g., epicardium/subepicardium) and near chambers (e.g., endocardium) would be separated first, followed by cells in the myocardium. Parts of cells in the myocardium might also be separated. The percentage of the epicardial cells using the 1 h whole-heart collagenase digestion protocol should be higher than that in the entire heart.

At the external and internal parts of the hearts, the expression of cardiogenesis genes *GATA4* and *Nkx2.5* significantly increased following MI. *GATA4* expression peaked on day 11 (15.00 ± 13.24 on day 11 vs 1.00 ± 0.35 pre MI; p = 0.029), and *Nkx2.5* expression peaked on day 21 after MI (9.74 ± 4.73 on day 21 vs 1.00 ± 0.91 pre MI; p = 0.002) (Figure 1B). At the myocardial part of the hearts, alternations in gene expression were vague (Figure 1B, lower panels) (unit: expression fold over no MI).

### Cardiomyogenic progenitor cells exist in the postnatal mammalian heart

The increased expression of cardiogenesis genes (i.e., *GATA4, Nkx2.5*) after myocardial injury hinted that cardiac lineage-specific progenitor cells existed in the postnatal heart. *Nkx2.5* is one of the earliest transcription factors expressed during embryonic cardiogenesis. A 2.1 kilobase enhancer located 9.5 kilobase upstream of the translation start of murine *Nkx2.5* along with a 500 base-pair *Nkx2.5* base promoter was used to generate cardiac-specific Nkx2.5 enh-eGFP mice, in which eGFP is expressed specifically in Nkx2.5 cardiac progenitor cells in the developing hearts [18,29]. The mice were used to examine if cardiomyogenic progenitor cells exist in the postnatal mammalian heart (Supplementary Figure 1). Nkx2.5 enh-eGFP^positive^ cells were isolated (Figure 2A, left panel) from the hearts of postnatal Nkx2.5 enh-eGFP mice, and cultured in differentiation medium [18] to test the cardiomyogenic potential of postnatal Nkx2.5 enh-eGFP^positive^ cells. The isolated Nkx2.5 enh-eGFP^positive^ cells did not express cTnI and striation in the first 2 days after sorting (Figure 2A, middle panel). After single culture for 10 days, Nkx2.5 enh-eGFP^positive^ cells differentiated into cells with cardiomyocyte-like phenotype expressing cTnI and striation (Figure 2A, right panel), suggesting that postnatal Nkx2.5 enh-eGFP^positive^ cells are cardiomyogenic progenitor cells. Nkx2.5 enh-eGFP^positive^ cells sorted 1 week following experimental MI stained positive for precardiac mesoderm marker (GATA4) (Figure 2B, a) and cardiac precursor marker (Nkx2.5) (Figure 2B, b), suggesting they are cardiac progenitor cells. The Nkx2.5 enh-eGFP^positive^ cells presented with small round cells and were primarily located at the outer layer of compact myocardium just beneath the epicardium (**Supplementary Figure 2**).

### *In vivo* evidence that Nkx2.5 enhancer-expressing cells differentiate into cardiomyocytes

To confirm if Nkx2.5 enhancer-expressing cells differentiate into cardiomyocytes *in vivo*, we created an experimental MI by ligating the left anterior descending coronary artery of 6–8 week-old inducible Nkx2.5 enh-Cre/R26R-LacZ mice that express Cre under the control of both Nkx2.5 cardiac enhancer and the tetracycline transactivator using tet-off system [20] with R26R-LacZ as a reporter. Doxycycline was administered from conception to MI. There was no LacZ+ staining cells in the unoperated group, indicating no significant leakage of the tet-off system (Figure 3A, a). Six weeks following MI, the descendent cells of post-MI Nkx2.5 enhancer-expressing cells were identified by LacZ staining (Figure 3A, b–d). These galactosidase+ cells differentiated into striated cardiomyocytes (labeled by cTnT) (Figure 3B), accounting for 3% of all cardiomyocytes. The lineage tracing result provides direct evidence that Nkx2.5 enhancer-expressing cells are cardiomyogenic progenitor cells.

### Activation of Nkx2.5 enhancer-expressing cells after myocardial injury

To determine if cardiac injury activates cardiac progenitor cells, we created an experimental MI by ligating the left anterior descending coronary artery of 6–8 week-old Nkx2.5 enh-eGFP mice and analyzed the percentage of intracardiac eGFP+ cells by flow cytometry 1, 3, 5, 7, 11, 14 and 21 days following MI (Figure 4A). The baseline cell percentage of eGFP+ cells (pre-MI) was low (0.06% ± 0.064%) (Figure 4B & C). The Nkx2.5 enh-eGFP^positive^ cell percentage increased markedly after experimental MI and peaked 2 weeks post-MI: pre-MI, 0.06% ± 0.064%; Day 1, 0.07% ± 0.049%; Day 3, 0.14% ± 0.010%; Day 5, 0.62% ± 0.650%; Day 7, 0.73% ± 0.333% (p = 0.004 compared with pre-MI; p = 0.028 compared with Day 1); Day 11, 0.75% ± 0.277% (p = 0.001 compared with pre-MI; p = 0.010 compared with Day 1; p = 0.043 compared with Day 3); Day 14, 0.86% ± 0.077% (p < 0.001 compared with pre-MI; p < 0.001 compared with Day 1; p = 0.001 compared with Day 3); Day 21, 0.63% ± 0.688% (n = 4 to 5 in each group) (Figure 4B & C). Collectively, the number of Nkx2.5 enh-eGFP^positive^ cells increased after MI and expanded up to 14-fold 2 weeks post-MI. The activation of these cardiac progenitor cells suggests that they might contribute to heart repair.

### The developmental origin of postnatal cardiac progenitor cells

Although it has been proposed that a stem cell pool might contribute to postnatal cardiomyocyte renewal in mammals [4], the origin of such cells remains uncertain.

To define the developmental source of postnatal cardiac progenitor cells, a retrospective Cre-recombinase lineage analysis was designed using a series of triple transgenic mice carrying the Nkx2.5 enh-eGFP construct, variant lineage-specific Cre (endothelial cells: Tie2-Cre, neural crest cells: Pax3-Cre, cardiomyocytes: α-MHC-MerCreMer, epicardium: GATA5-Cre, Wt1^CreERT2^), and the R26R-LacZ reporter line.

An experimental MI was then created on different lineage-specific Cre±/Nkx2.5 enh-eGFP/R26R-LacZ± mice, the hearts were harvested, digested and sorted for the Nkx2.5 enh-eGFP^positive^ cells 1 week post-MI. Cellular DNA of eGFP^positive^ cells and unsorted cells were isolated. PCR was performed to assess Cre-mediated excision to trace the origin of activated postnatal Nkx2.5 enh-eGFP^positive^ cells (Figure 5A). The negative excision bands of the sorted Nkx2.5 enh-eGFP^positive^ cells from the Tie2-Cre and Pax3-Cre series confirmed that the activated Nkx2.5 enh-eGFP^positive^ cells following MI do not originate from endothelial cells and cardiac neural crest cells (Figure 5B & C).

To further confirm that the activated Nkx2.5 enh-eGFP^positive^ cells do not arise from the pre-existing cardiomyocytes, the pre-existing cardiomyocytes were labeled with β-galactosidase+ with 4-OH tamoxifen intraperitoneal injection into the αMHC-MerCreMer/Nkx2.5 enh-eGFP/R26R-LacZ mice at a dose of 2 mg three-times a week for 2 weeks (i.e., a total of 12 mg) prior to MI. That study confirmed that the activated Nkx2.5 enh-eGFP^positive^ cells did not originate from the pre-existing mature cardiomyocytes because the isolated Nkx2.5 enh-eGFP^positive^ cells did not show excision (n = 7) (Figure 5D). The result suggested that the cardiomyocytes do not undergo Nkx2.5 fetal gene re-expression, nor do they dedifferentiate into Nkx2.5 enh-eGFP^positive^ cells.

The positive excision PCR of the activated Nkx2.5 enh-eGFP^positive^ cells from the hearts of epicardial cell lineage, GATA5-Cre/Nkx2.5 enh-eGFP/R26R-LacZ mice suggested the epicardial origin of postnatal Nkx2.5 cardiac progenitor cells (Figure 6A). The possibility of aberrant expression of LacZ was ruled out by negative excision of the sorted Nkx2.5 enh-eGFP^positive^ cells from Nkx2.5 enh-eGFP, GATA5-Cre/Nkx2.5 enh-eGFP and Nkx2.5 enh-eGFP/R26R-LacZ mice (Figure 6A).

These assays revealed that the postnatal cardiac progenitor cells arose from epicardial cells, not from endothelial cells, cardiac neural crest cells, or pre-existing cardiomyocytes.

### Embryonic epicardium: developmental origin of postnatal cardiac progenitor cells

Gata5-Cre lineage analysis suggested an epicardial origin of postnatal Nkx2.5 cardiac-lineage progenitor cells; however, the origin could be adult, perinatal or embryonic epicardial cells. To determine the cell origin of the postnatal progenitors, the inducible epicardial lineage, Wt1^CreERT2^/Nkx2.5 enh-eGFP/R26R-LacZ mice were generated and the Wt1 cells were labeled with 4-OH tamoxifen at different developmental stages.

The peri-MI Wt1 epicardial cells were labeled with 4-OH tamoxifen intraperitoneal injection at a dose of 2 mg three-times a week for 1 week prior to MI and three-times a week for 1 week after MI (i.e., a total of 12 mg). The PCR results showed that the activated Nkx2.5 enh-eGFP^positive^ cells did not derive from the peri-MI Wt1 epicardial cells as the sorted GFP+ cells showed negative results (Figure 6B). The unsorted cells showed negative PCR result (Figure 6B). LacZ staining of the heart sections from the Wt1^CreERT2^/R26R-LacZ mice, which had been labeled peri-MI Wt1 cells following 4-OH tamoxifen injection before and after MI, also revealed no β-galactosidase positive cells (Figure 7A). Together, the PCR and LacZ staining results confirmed that no or very few adult Wt1 epicardial cells existed even following MI.

The perinatal Wt1 epicardial cells were labeled with 4-OH tamoxifen injection on ED 18.5 (Figure 6C). The negative PCR results revealed that the activated Nkx2.5 progenitor cells did not come from perinatal Wt1 epicardial cells (Figure 6C).

The embryonic Wt1 cells were subsequently labeled with 4-OH tamoxifen on ED 10.5, and the positive excision PCR confirmed the embryonic epicardial origin of postnatal Nkx2.5 cardiac progenitor cells (Figure 6D). LacZ staining of the heart sections from Wt1^CreERT2^/R26R-LacZ mice, in which the embryonic Wt1 cells were labeled with 4-OH tamoxifen on ED 10.5, showed that embryonic epicardium-derived cells were present in both the epicardium and myocardium of the adult heart (Figure 7B).

Taken together, GATA5-Cre and inducible Wt1-Cre (Wt1^CreERT2^) lineage tracing confirmed the embryonic epicardial origin of the postnatal cardiac progenitor cells.

## Discussion

In recent years, evidence supporting the existence of cardiomyocyte renewal in postnatal mammalian hearts has been mounting [3,5,6]. However, the origin of the cells contributing to postnatal cardiomyogenesis remained uncertain. We confirmed that Nkx2.5 enhancer-expressing cells are present in postnatal hearts and expand remarkably following myocardial injury. Using inducible Nkx2.5 enh-Cre/R26R-LacZ mice [20] to lineage trace postnatal Nkx2.5 enhancer-expressing cells, we confirmed that Nkx2.5 enhancer-expressing cells contribute directly to postnatal cardiomyogenesis after myocardial injury. These studies determined the cardiomyogenic differentiation potential of those Nkx2.5 enhancer-expressing cells both *in vitro* and *in vivo*. Lineage tracing studies further confirmed those cardiomyogenic progenitor cells were indeed embryonic epicardium-derived cells.

In the current study, the number of Nkx2.5 enhancer-expressing cells was low in the postnatal heart, but was activated following cardiac injury. The reactivation of the Nkx2.5 progenitor cells suggested that they might help to repair or regenerate the injured myocardium.

Postnatal cardiomyocytes reportedly dedifferentiate following cardiac injury and re-express markers of embryonic cardiomyocytes [30–32]. Although the increased expression of the cardiogenesis genes *GATA4* and *Nkx2.5* after MI could be due to the result of either dedifferentiation or fetal gene re-expression of the cardiomyocytes, we believe that is unlikely to be the case because the pre-existing cardiomyocytes did not undergo *Nkx2.5* re-expression after MI (Figure 5D).

Embryonic epicardial progenitor cells, marked by Tbx18 or Wt1, reportedly contribute to embryonic cardiomyogenesis [25,33]. A recent study indicated that Wt1+ cells, via priming by thymosin-β4, might transdifferentiate into cardiomyocytes following cardiac injury in a mouse model [12]. Those authors found that the adult heart can respond to injury with modest increases in Wt1 progenitors but without initiating a cardiogenic program [12]. The data generated in this study showed that postnatal Nkx2.5 progenitor cells arise from embryonic Wt1 epicardial cells, but not from adult Wt1 epicardial cells. The adult Wt1 epicardial progenitor cells described by Smart *et al*. seemed different from the postnatal Nkx2.5 progenitor cells. Further, Chong *et al*. described a population of adult cardiac-resident mesenchymal stem cell-like stem cells (cardiac colony-forming-unit fibroblasts; cCFU-Fs) with the expression of PDGF-α exhibited multipotency including cardiomyocytes, endothelial cells, smooth muscle cells, bone, cartilage, adipose tissue, etc. [13]. Those authors also confirmed the epicardial origin of the cCFU-Fs. The cCFU-Fs also seemed different from Nkx2.5 cardiac progenitor cells because they did not express Nkx2.5 [13]. Further research will be required to determine whether cCFU-Fs and postnatal Nkx2.5 progenitor cells represent hierarchically related progenitors.

Using GATA5-Cre and Wt1^CreERT2^ lineage mice, the lineage tracing performed in this study confirmed the epicardial origin of postnatal Nkx2.5 progenitor cells. However, GATA5-Cre recombinase activity is not only expressed in epicardium. Instead, GATA5-Cre recombinase is also expressed in the cavities and their linings, the hepatobiliary system, mesenchyme, renal and urinary system (based on information on the Mouse Genome Informatics Website). Tamoxifen-inducible Wt1^CreERT2^ mouse line was reported inefficiently to recombine the epicardium and its cellular derivatives [34]. Our initial tests revealed that injection of high-dose 4-OH tamoxifen (e.g., 1.5 mg or more) led to embryonic death. We thus labeled the embryonic Wt1 cells with low dose (e.g., 1 mg) 4-OH tamoxifen on ED 10.5, which might resulted in low recombination efficiency obtained with the tamoxifen-inducible Wt1^CreERT2^ line in epicardial cells. Even so Wt1^CreERT2^ recombinase activity is still detected in the postnatal Nkx2.5 progenitor cells, strongly suggests the embryonic epicardial origin of postnatal Nkx2.5 progenitor cells.

The discovery of cardiogenesis gene expression in the external and internal part of the heart (Figure 1B) and additional lineage tracing using GATA5-Cre and Wt1^CreERT2^ line (Figure 7) strongly suggest an epicardial origin for postnatal Nkx2.5 cardiac progenitor cells.

Our study identifies Nkx2.5 enhancer-expressing cells as a source for postnatal cardiomyogenesis. However, the results cannot exclude the possibility that postnatal cardiac regeneration occurs through cardiomyocyte proliferation.

## Conclusion

The presented study demonstrated that Nkx2.5 cardiomyogenic progenitor cells existed in the postnatal mammalian heart and originated from the embryonic epicardium.

## Future perspective

The major challenge in cardiovascular medicine is the inability to replace the large number of cardiomyocytes lost after cardiac injury. This study demonstrates the cell type and the origin of the cells involved in postnatal cardiomyogenesis. These results will facilitate cell therapy for cardiac regeneration, the pharmacological targeting of the regenerating cells, enhancing endogenous cardiac regeneration and further understanding the mechanisms of cardiovascular diseases.

**Table 1.** **Categories of the genetically manipulated mice and their purposes.**

**Genetically manipulated mice**	**Purposes**
Nkx2.5 enh-eGFP	To label Nkx2.5 cardiac progenitor cells
Inducible Nkx2.5 enh-Cre	To lineage-trace postnatal cardiac progenitor cells and their derivatives
R26R-LacZ	As a reporter
Tie2-Cre	Endothelial cell lineage. Used in lineage-specific Cre/Nkx2.5 enh-eGFP/R26R-LacZ mice to trace the developmental origin of postnatal cardiac progenitors
Pax3-Cre	Neural crest cell lineage. Used in lineage-specific Cre/Nkx2.5 enh-eGFP/R26R-LacZ mice to trace the developmental origin of postnatal cardiac progenitors
αMHC-MerCreMer	Inducible cardiomyocyte lineage. Used in lineage-specific Cre/Nkx2.5 enh-eGFP/R26R-LacZ mice to trace the developmental origin of postnatal cardiac progenitors
GATA5-Cre	Epicardial cell lineage. Used in lineage-specific Cre/Nkx2.5 enh-eGFP/R26R-LacZ mice to trace the developmental origin of postnatal cardiac progenitors.
Wt1^CreERT2^	Inducible Wt1 epicardial cell lineage. Used in lineage-specific Cre/Nkx2.5 enh-eGFP/R26R-LacZ mice to trace the developmental origin of postnatal cardiac progenitors by labeling variant stages of epicardial Wt1 cells

**Executive summary**

Myocardial injury triggers the expression of embryonic cardiogenesis genes.
Using Nkx2.5 enhancer-eGFP (Nkx2.5 enh-eGFP) mice, the authors confirmed that Nkx2.5 enhancer-expressing cells existed in the postnatal mouse heart and could differentiate into striated cardiomyocytes.
Nkx2.5-eGFP+ cells increased remarkably after experimental myocardial infarction (MI).
*In vivo* lineage tracing study using inducible Nkx2.5 enhancer-Cre (inducible Nkx2.5 enh-Cre)/ROSA26 reporter mice documented the cardiomyogenesis fate of these activated cardiac progenitor cells.
The authors traced the developmental origin of postnatal Nkx2.5 cardiac progenitor cells by analyzing Cre excision of activated Nkx2.5-eGFP+ cells from different lineage-Cre/Nkx2.5 enh-eGFP/ROSA26 reporter mice.
Post-MI Nkx2.5-eGFP+ cells originated from the embryonic epicardial cells, not from the pre-existing cardiomyocytes, endothelial cells, cardiac neural crest cells, or perinatal/postnatal epicardial cells.
